# Influence of weight status in the response to Step-2 maintenance therapies in children with asthma

**DOI:** 10.1136/bmjresp-2019-000401

**Published:** 2019-04-11

**Authors:** Cristina Longo, Gillian Bartlett, Tibor Schuster, Francine M. Ducharme, Brenda MacGibbon, Tracie A. Barnett

**Affiliations:** 1 Family Medicine, McGill University, Montreal, Québec, Canada; 2 Respiratory Medicine, Academic Medical Center, Amsterdam, The Netherlands; 3 Pediatrics and Social and Preventive Medicine, Université de Montréal, Montreal, Québec, Canada; 4 Pediatrics, Centre de recherche du CHU Sainte-Justine, Montreal, Québec, Canada; 5 Mathematics, UQAM, Montreal, Québec, Canada; 6 Epidemiology and Biostatistics, INRS-Institut Armand-Frappier, Laval, Québec, Canada

**Keywords:** asthma, BMI, children, inhaled corticosteroids, leukotriene receptor antagonists, management failure, weight status, obesity

## Abstract

**Introduction:**

Overweight children with asthma may display impaired response to inhaled corticosteroids (ICS), possibly due to non-eosinophilic inflammation or weight-related lung compression; these mechanisms may differentially affect response to ICS and leukotriene receptor antagonists (LTRAs). We assessed whether weight status modified the response to low-dose ICS and LTRA Step-2 monotherapy.

**Methods:**

A historical cohort study from clinical data linked to administrative databases was conducted among children aged 2–18 years with specialist-diagnosed asthma who were initiating or continuing a Step-2 monotherapy from 2000 to 2007 at the Montreal Children’s Hospital Asthma Centre. The outcome was time-to-management failure defined as any step-up in therapy, acute care visit, hospitalisation or oral corticosteroids for asthma, whichever occurred first. The independent and joint effects of weight status (body mass index [BMI] percentile) and time-varying treatment on time-to-management failure were estimated with marginal structural Cox models. The likelihood ratio test (LRT) and relative excess risk due to interaction (RERI) were computed to assess treatment effect modification by weight status on the multiplicative and additive scales.

**Results:**

Of the 433 and 85 visits with a low-dose ICS and LTRA prescription, respectively, 388 management failures occurred over 14 529 visit-weeks of follow-up. Children using LTRA compared with low-dose ICS tended to have an overall higher risk of early management failure (HR 1.52; 95% CI 0.72 to 3.22). Irrespective of treatment, the hazard of management failure increased by 5% (HR 1.05; 95% CI 1.01 to 1.10) for every 10-unit increase in BMI percentile. An additional hazard reduction of 17% (HR 0.83; 95% CI 0.70 to 0.99) was observed for every 10-unit increase in BMI percentile among LTRA users, but not for ICS (HR 0.95; 95% CI 0.86 to 1.04). The LRT indicated a departure from exact multiplicativity (p<0.0001), and the RERIs for ICS and LTRA were −0.05 (95% CI −0.14 to 0.05) and −0.52 (95% CI −1.76 to 0.71).

**Conclusions:**

Weight status was associated with earlier time-to-management failure in children prescribed Step-2 therapy. This hypothesis-generating study suggests that LTRA response increases in children with higher BMI percentiles, although further research is warranted to confirm findings.

**Key messages**

- Children with the obese-asthma phenotype may exhibit higher degrees of airflow obstruction and non-eosinophilic inflammation, which may respond more favourably to leukotriene receptor antagonists (LTRAs), than inhaled corticosteroids (ICS), due to their different modes of action; however, this has not yet been studied in children.
- Children with excess weight were more likely to have an early management failure event (step-up in therapy or exacerbation) and respond to LTRA, but not low-dose ICS, when compared with their normal-weight counterparts.
- These data support LTRA as a possible alternative therapy for mild asthma in children with excess weight who may have not previously responded to low-dose ICS; further prospective research is warranted to confirm findings.

## Introduction

Over the last few decades, the prevalence of obesity in paediatric asthma has risen rapidly, with an estimated 20% and 30% of children with asthma reported to be overweight and obese, respectively.^1 2^ As its own distinct clinical phenotype,^3^ obese children with asthma have been shown to be exacerbation-prone and difficult to manage.^2 4–6^ As a result, these vulnerable children are often at a higher risk of school absenteeism^7^ and decreased quality of life^8^ when compared with their normal-weight counterparts.^4^ Along with increased morbidity, obese patients with asthma may have a suboptimal therapeutic response to inhaled corticosteroids (ICS),^6 9 10^ which constitute the current gold standard for asthma management in children.^11^

Poor response to ICS among obese patients has been hypothesised to be due to altered lung function and inflammatory processes.^4 12–14^ The excess weight-induced mechanical stress placed on the thoracic cage can lead to higher degrees of airflow limitation, which some speculate may lead to reduced peripheral lung deposition of inhaled therapies.^9 13^ There is also support for an alternative inflammatory process in obese-asthma that is independent of the classical Th2 eosinophilic-mediated pathway,^14–16^ with eosinophilic phenotypes known to be corticosteroid-responsive in patients with asthma without refractory disease. As the obese-asthma inflammatory pathway may be mediated by a neutrophilic mechanism,^17^ experts have questioned the applicability of the current treatment guidelines to patients with excess weight,^18^ recommending low-dose ICS as the first-line Step-2 maintenance therapy for mild persistent asthma.^19 20^ Nevertheless, findings supporting a differential response attributed to weight status in children have yet to be confirmed.

Leukotriene receptor antagonists (LTRAs) are a second-line Step-2 oral maintenance monotherapy that inhibit both eosinophil-mediated and neutrophil-mediated inflammation and whose action is independent of lung mechanics to achieve therapeutic targets; it may thus serve as an alternative therapy in patients with excess weight.^21–23^ Given the paucity of studies evaluating the comparative effectiveness of LTRA monotherapies by weight status in children with asthma, low-dose ICS remains the preferred first-line Step-2 therapy across guidelines. Thus, the potential benefits of LTRA versus ICS in terms of achieving control and preventing exacerbation in children with excess weight have not been established.

The primary objective of this study was to assess if weight status modifies the response to low-dose ICS and LTRA monotherapy, specifically by evaluating whether the association between Step-2 therapies and time-to-management failure is modified by body mass index (BMI) percentile in children with asthma. We hypothesised that weight-related changes to lung physiology and mechanics would lead to a differential response to Step-2 therapies in children with higher BMI status.

## Methods

### Research design

A historical cohort study was conducted using a database that linked clinical chart data to Quebec administrative health and drug claims databases for the purposes of evaluating the effectiveness of different asthma maintenance therapies and risk factors for poor outcomes. The Paediatric Asthma Database contained 15 147 clinical records of 4621 children who visited the Asthma Centre of the Montreal Children’s Hospital between January 2000 and December 2007.

### Patient and public involvement

Because this is a database study, the research question and outcomes measured were not informed by patients, nor were patients involved in the recruitment to or conduct of the study. As all data were anonymised, study results cannot be directly disseminated to the patients included in this study.

### Data sources

The Paediatric Asthma Database included detailed information on sociodemographics, asthma diagnosis, severity and control indicators as assessed by the consulting asthma specialist, environmental exposures, lung function test results and medical chart records of all prescribed asthma maintenance and/or rescue treatments, with the indication for medication use clearly labelled, for each visit at the Asthma Centre. Patient records from the Paediatric Asthma Database were linked to the hospital admission (MED-ECHO), Régie de l’assurancemaladie du Québec (RAMQ) medical service and prescription claims administrative databases. The RAMQ medical service database contains information on the type, location of service delivery and diagnostic codes using the International Classification of Diseases 9th and 10th revisions (ICD-9; ICD-10) for all medical services billed by physicians. The MED-ECHO database includes admission and discharge codes for all hospitalisations in Quebec. Children are provided universal access to all healthcare services in Quebec. Lastly, the RAMQ prescription claims database contains data on all drugs dispensed to patients enrolled in the Public Drug Insurance Plan (42% of Quebec residents).^24^

### Study population

Children were included if they were aged 2–18 years, consulted the Asthma Centre during the study period 2000–2007, had an asthma diagnosis confirmed by a specialist, and were prescribed low-dose ICS or LTRA (Step-2) maintenance monotherapies as documented in the medical chart at the index visit date. Patients were excluded if they were diagnosed with bronchopulmonary dysplasia or cystic fibrosis or were not enrolled in the public drug or health insurance plan at least 6 months prior to and 3 months following the visit date. The study population included children who were initiating or continuing a prescription of low-dose ICS or LTRA. Children initiating (‘incident’ users) and continuing therapy (‘prevalent’ users) were defined as those who had no claims and at least one claim for low-dose ICS or LTRA, respectively, in the 6 months preceding the index date (‘lookback period’). Children who had claims for medium-dose or high-dose ICS or ICS in combination with long-acting beta-2 agonists (LABAs) or LTRA during the lookback period were excluded. The cohort entry (index) date was defined as the date at which patients met all inclusion criteria. We included all visit dates eligible for cohort entry based on the above criteria; thus, some children contributed multiple entry dates (also known as ‘trials’^25^) to the cohort and thus could contribute data to both ICS and to LTRA at different points in time. Follow-up ended at the outcome date, end of insurance coverage, 1-year post index date or 31 December 2007, whichever occurred first.

### Outcome

Management failure was defined as a composite endpoint that included any of the following events: (1) a step-up in maintenance therapy, that is, higher dose ICS, ICS/LABA or ICS/LTRA combination therapy dispensed during follow-up; (2) a short course of oral corticosteroids; (3) an acute care visit; or (4) a hospital admission for an asthma exacerbation (ICD-9 493.X; ICD-10 J45.X). We instated a lag of 3 days following cohort entry before assessing the outcome to exclude the possibility of pre-existing exacerbations.

### Exposures

#### Weight status: BMI percentile

BMI percentiles were computed using Z-scores with age- and sex-specific WHO growth reference values from the height and weight measurements documented by trained healthcare professionals at the Asthma Centre index visit.^26^

#### Treatment

A time-varying exposure definition was implemented to classify Step-2 maintenance therapy use throughout follow-up. Children dispensed low-dose ICS, that is, <200 μg/day (<250 μg/day if ≥12 years old) of hydrofluoroalkane-propelled beclomethasone dipropionate or equivalent measured ex-valve,^20^ were classified as exposed to low-dose ICS monotherapy for the duration of the claim. Children dispensed LTRA (montelukast or zafirlukast) were classified as exposed to LTRA monotherapy for the duration of the claim. If the ICS daily dose documented in the claims database was discordant with Asthma Centre records, the expected duration of the drug claim was adjusted to better reflect the prescribed daily dose as documented in the medical charts at the index date. If a child held an active prescription claim for a Step-2 maintenance therapy at the index date, he/she was considered exposed from cohort entry until the expected completion date of that pre-existing drug claim. Gaps of no therapy, that is, the time defined by the absence of claims for both low-dose ICS and LTRA, were classified as periods of ‘treatment non-compliance’, which served as a reference group.

### Time-fixed and time-varying confounders

Sociodemographic variables (age, sex, ethnicity and average family income estimated from census data using six-digit postal codes), user type (incident vs prevalent), asthma phenotype (persistent vs non-persistent), respiratory comorbidities (including atopy, recurrent otitis, sinusitis, pneumonia, gastro-oesophageal reflux, obstructive sleep apnoea, bronchopulmonary aspergillosis, vocal cord dysfunction or dysphagia), exposure to cigarette smoke and morbidity (number of exacerbations in the preceding year) were documented at the index visit and defined as time-fixed confounders. Time-varying confounders included physicians’ global assessment of asthma severity and prebronchodilator percent predicted forced expiratory volume in 1 s (FEV_1_) test values as they were updated at each visit to the Asthma Centre. In some children, higher dose ICS was prescribed as a rescue therapy by the Asthma Centre physician, which could have confounded treatment effects. Thus, we also controlled for higher dose ICS rescue therapy use, where the child was classified as using the rescue therapy from the date at which he/she claimed the rescue medication (as prescribed in medical charts) until the end of follow-up, and season to account for viral-induced and allergy-induced worsening of asthma symptoms.

### Statistical analysis

We used multiple imputations to address missing data for BMI percentile (1.2%), ethnicity (9.3%), percent predicted FEV_1_ (13.1%), exposure to smoking (22.6%) and family income (22.9%) at the index date. We did not test differences in baseline characteristics between treatment groups, as recommended by the Strengthening the Reporting of Observational Studies in Epidemiology guidelines. We fit extended Cox models to obtain conditional associations for Step-2 maintenance therapies and a 10-unit increase in BMI percentile at baseline with respect to the hazard of management failure, while adjusting for all measured confounders. We also fit marginal structural Cox models for time-varying treatments, which have the added advantage of correcting for time-dependent confounding.^27^ To correct for possible selection bias potentially induced by time-dependent confounding and losses to follow-up, we fit two separate propensity score models, for treatment use and censoring, respectively, at each follow-up week interval, conditional on all measured confounders and covariates. We then computed stabilised inverse probability of treatment and censoring weights for each follow-up week interval.^27 28^ We used the product of these weights in the estimation of the final marginal HRs for treatment.^27 28^ In the marginal model, the weighted approach enables the estimation of an HR that compares the marginal hazards of time-to-management failure between treatment categories (non-compliant, low-dose ICS or LTRA) had all patients been consistently taking their LTRA monotherapy, their low-dose ICS monotherapy or been non-compliant (ie, not exposed to any treatment), throughout the follow-up period. If the propensity score model is correctly specified, the marginal structural model emulates a randomised controlled trial contrasting the expected time-to-event outcomes of each treatment arm (no treatment, low-dose ICS and LTRA monotherapy) had all patients remained in their assigned arm throughout follow-up—an estimate that reflects what would happen in an ideal setting (efficacy).^29 30^ Conversely, the conditional model estimates an HR comparing the hazards of time-to-management failure between patients exposed to ICS, LTRA or non-compliance at observed event times T, given patients’ observed treatment (patterns of use) and covariate histories, as well as having remained under observation until time T—an estimate that is reflective of real life (effectiveness).

Violations to the proportional hazards assumption were investigated by visually inspecting the scaled Schoenfeld residuals plot and confirmed with the likelihood ratio test (LRT) for improved fit when an interaction term with time was included in the model. To obtain conditional and marginal treatment HRs for different weight status categories, interaction terms between treatment and BMI percentile at baseline was included. When evaluating whether treatment effects were modified by BMI percentile, we computed the relative excess risk due to interaction (RERI)^31^ and the LRT for the marginal and conditional models to assess interaction on the additive and multiplicative scales, respectively.

Based on the data distribution, BMI percentiles were classified ad hoc into normal (BMI ≤80th percentile) and excess weight (BMI >80th percentile) using the median as the cut-off point to improve statistical power; this dichotomisation was used to estimate joint effects and the specific survival curves for each Step-2 therapy and weight category combination, as well as the treatment (ICS or LTRA) HRs stratified by weight category. Sensitivity analyses were conducted to verify (1) potential non-linear relationships between BMI percentile and treatment effects with restricted cubic splines; (2) effects observed only in those with persistent asthma; and (3) the robustness of methods for handling missing data (multiple imputations vs complete case analysis). In addition, subgroup analyses explored whether findings differed in incident and prevalent users. Robust SEs were used to account for the inclusion of multiple ‘person-visits’ and the weights in the marginal model. Data management was performed with SAS V.9.4, and survival curves were illustrated using Stata Statistical Software V.13, while other analyses were performed using RStudio V.1.0.44.

## Results

**Figure 1:**
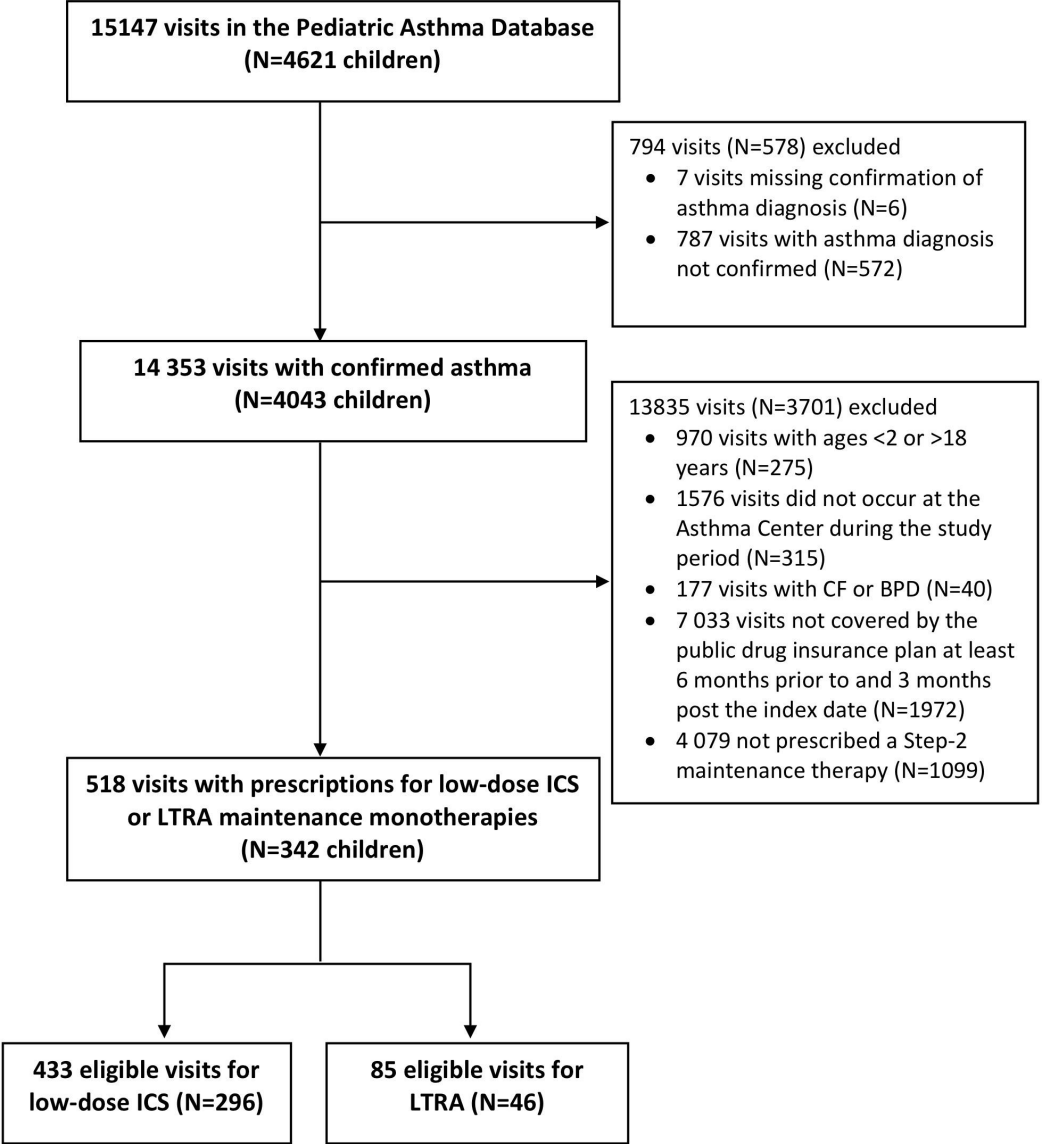
Study population flow chart. CF, cystic fibrosis; BPD, bonchopulmonary dysplasia; ICS, inhaled corticosteroids; LTRA, leukotriene receptor antagonist.

Of the total 15 147 visits (N=4621 patients) in the Paediatric Asthma Database, 518 (n=342) person-trials were included in the final cohort (figure 1), of whom 85 and 433 were prescribed LTRA and low-dose ICS at the index visit, respectively. The number of visits contributed by each patient is illustrated in online supplemental figure e-1.

10.1136/bmjresp-2019-000401.supp1 Supplementary data

Children included in the cohort were mostly male (56%), aged 5 years and older (87.5%), with a mean treatment period of 11 weeks (ranging from 0 to 52 weeks). Children prescribed low-dose ICS had more moderate-severe asthma and comorbidities, as well as higher healthcare utilisation in the previous year, than those prescribed LTRA. On average, children prescribed LTRA were older, had higher family income, and reported more exercise-triggered symptoms compared with those prescribed low-dose ICS (table 1).

**Table 1:** Baseline characteristics of incident and prevalent users prescribed Step-2 maintenance therapy

Baseline characteristics	LTRA (n*=85)	ICS (n*=433)
Age,$\bar{x}\pm \sigma$	11.3 ± 3.9	9.7±4.2
	Female, n (%)	42 (49.4)	186 (43.0)
Ethnicity, n (%)		
Caucasian	68 (80.0)	261 (60.3)
Black	8 (9.4)	64 (14.8)
Other	9 (10.6)	108 (24.9)
Income, M±IQR	45 684 (19 929)	40 187 (19 527)
	Weight status, n (%)		
BMI ≤80th percentile	44 (51.8)	218 (50.4)
BMI >80th percentile	41 (48.2)	215 (49.6)
Exposure to cigarette smoke, n (%)	41 (48.2)	121 (27.9)
% Predicted FEV_1_,$\bar{x}\pm \sigma$	99±12.8	98±14.1
	Asthma phenotype, n (%)		
Episodic/Seasonal	37 (43.5)	174 (40.2)
Persistent	48 (56.5)	259 (59.8)
Global assessment of severity, n (%)		
Mild	84 (98.8)	340 (78.5)
Moderate-severe	1 (1.2)	93 (21.5)
Exacerbations for asthma in previous year, n (%)		
Acute care visits		
0	15 (28.6)	64 (14.78)
1	18 (20.0)	99 (22.86)
2	22 (31.4)	104 (24.01)
≥3	30 (20.0)	142 (32.8)
Hospitalisations		
0	83 (97.7)	368 (85.0)
≥1	2 (2.3)	65 (15.0)
Oral corticosteroids		
0	79 (94.3)	298 (68.8)
≥1	6 (5.7)	135 (31.2)
Asthma-related comorbidities, n (%)†		
None	55 (64.7)	202 (46.7)
Atopy‡	26 (30.6)	214 (49.4)
Upper/Lower respiratory conditions§	7 (8.2)	43 (9.9)
Triggers, n (%)†		
Viral	54 (63.5)	310 (71.6)
Allergic	38 (44.7)	191 (44.1)
Effort	53 (62.4)	189 (42.7)
Temperature	7 (8.2)	66 (14.9)

*Number of person-trials.

†Categories are not mutually exclusive.

‡Atopic conditions comprise eczema, allergic rhinitis, conjunctivitis and food allergies.

§Chronic upper or lower respiratory tract comorbidities include a history of recurrent otitis, sinusitis, pneumonia, gastro-oesophageal reflux disease, obstructive sleep apnoea, bronchopulmonary aspergillosis, vocal cord dysfunction or dysphagia.

BMI, body mass index; FEV_1_, forced expiratory volume in 1 s; ICS, inhaled corticosteroids; LTRA, leukotriene receptor antagonist.

There were a total of 338 management failures during the 14 529 trial-weeks of follow-up. The incidence rates for management failure remained relatively stable between prescribed treatment at the index visit (ICS vs LTRA), regardless of the number of visits contributed by the child in the analysis; however, the incidence rate was elevated for children using LTRA during follow-up who contributed two or more visits compared with those contributing a unique visit (online supplemental tables e-1a and e-1b). In the main effects model, the conditional and marginal hazard of management failure increased by 6% (HR 1.06; 95% CI 1.01 to 1.10) and 5% (HR 1.05; 95% CI 1.01 to 1.10) for every 10-unit increase in BMI percentile, respectively (table 2). While not statistically different, results suggested a trend towards an overall poorer response to LTRA than to low-dose ICS (conditional HR 1.15, 95% CI 0.68 to 1.61; marginal HR 1.52, 95% CI 0.72 to 3.22) with respect to time-to-management failure.

10.1136/bmjresp-2019-000401.supp2 Supplementary data

**Table 2:** Conditional and marginal model estimates for time-to-management failure

Models	Average HR (95% CI)*	
	Conditional	Marginal
**Main** **e** **ffects** **m** **odel**		
BMI percentile (for every 10 units)	1.06 (1.01 to 1.10)	1.05 (1.01 to 1.10)
ICS vs treatment non-compliance	0.91 (0.69 to 1.20)	0.93 (0.70 to 1.23)
LTRA vs treatment non-compliance	1.05 (0.68 to 1.61)	1.11 (0.72 to 1.70)
LTRA vs ICS	1.15 (0.74 to 1.80)	1.52 (0.72 to 3.22)
**Model with** **i** **nteraction** **t** **erm**		
BMI percentile (for every 10 units)	1.09 (1.03 to 1.16)	1.08 (1.02 to 1.15)
ICS vs treatment non-compliance	1.45 (0.66 to 3.16)	1.37 (0.61 to 3.10)
LTRA vs treatment non-compliance	7.94 (2.56 to 24.69)	4.62 (1.27 to 16.88)
ICS × BMI percentile	0.94 (0.85 to 1.03)	0.95 (0.86 to 1.04)
LTRA × BMI percentile	0.78 (0.67 to 0.91)	0.83 (0.70 to 0.99)
*LRT p* *value* *†*	<0.001	<0.001
*RERI ICS* *×* *BMI percentile* *‡*	−0.06 (−0.16 to 0.04)	−0.05 (−0.14 to 0.05)
*RERI LTRA* *×* *BMI percentile* *‡*	−1.29 (−3.55 to 0.97)	−0.52 (−1.76 to 0.71)

*After accounting for age, sex, ethnicity, income, user type, global assessment of severity score, number of exacerbations in previous year, exposure to smoke, asthma-related comorbidities, triggers, % predicted FEV_1_, ICS rescue use and season.

†LRT assesses improved goodness of fit when comparing nested models (the model with interaction terms vs the main effects model); a p value <0.05 indicates a statistically significant improved fit, that is, explaining a greater proportion of the variance in the outcome, and the likely presence of effect measure modification on the multiplicative scale.

‡RERI=HR_Therapy×BMI_ – HR_Therapy_ – HR_BMI_ +1; a negative RERI can be interpreted as the hazard reduction due to interaction on the additive scale (subadditivity), adjusted for measured confounders.

BMI, body mass index; FEV_1_, forced expiratory volume in 1 s; ICS, inhaled corticosteroids; LRT, likelihood ratio test; LTRA, leukotriene receptor antagonist; RERI, relative excess risk due to interaction.

In the conditional and marginal models that include an interaction term between Step-2 therapy and BMI percentile, a clinically relevant reduction in the risk of management failure was not observed with increasing BMI percentile among ICS users. In contrast, for children treated with LTRA, an additional 22% (HR 0.78; 95% CI 0.67 to 0.91) and 17% reduction (HR 0.83; 95% CI 0.70 to 0.99) in the conditional and marginal hazards of management failure were observed for every increase of 10 BMI percentile units, respectively. Of note, inclusion of the interaction term of treatment by BMI percentile significantly improved the fit (LRT p<0.001) in both models, indicating the presence of treatment effect modification on the multiplicative scale; the RERIs suggested a trend towards subadditivity, that is, the joint effects of BMI and treatment were less than the sum of their individual effects on the additive scale, which was most pronounced for LTRA (marginal RERI −0.52; 95% CI −1.76 to 0.71) relative to ICS (marginal RERI −0.05; 95% CI −0.14 to 0.05) (table 2). In the spline sensitivity analysis, a non-linear relationship between BMI percentile and the hazard of management failure was visually evident for ICS users, but not LTRA users, indicating that models assuming a linear relationship with BMI may overestimate the beneficial effect of ICS at an elevated weight status, suggesting a blunted response to ICS at higher BMI percentiles (online supplemental figure e-2).

The average conditional and marginal HRs for different treatment–weight status categories (≤80th vs >80th percentile) were also estimated in relation to a common reference group, namely normal-weight children who were non-compliant to treatment (table 3). Compared with this reference group, the marginal HRs of management failure for LTRA were 1.72 (95% CI 1.01 to 2.93) for normal-weight and 1.14 (95% CI 0.61 to 2.13) for excess-weight patients. For normal-weight and excess-weight children on ICS, the estimates were 0.96 (95% CI 0.62 to 1.48) and 1.21 (95% CI 0.84 to 1.74), respectively, suggesting a trend towards a reduced response to ICS with higher BMI. Children with excess weight who were also non-compliant to treatment had a marginal HR of 1.38 (95% CI 1.00 to 1.89). The conditional model demonstrated similar trends (table 3).

**Table 3:** Conditional and marginal joint effects of treatment and weight status on time-to-management failure

Treatment	Weight status	Events/Visit-weeks (n)	Average HR (95% CI)*	
			Conditional	Marginal
LTRA monotherapy	BMI >80th percentile	12/563	1.09 (0.60 to 2.00)	1.14 (0.61 to 2.13)
	BMI ≤80th percentile	16/470	1.69 (1.00 to 2.88)	1.72 (1.01 to 2.93)
	Overall	28/1033		
Low-dose ICS monotherapy	BMI >80th percentile	59/2184	1.18 (0.83 to 1.69)	1.21 (0.84 to 1.74)
	BMI ≤80th percentile	61/2771	0.92 (0.60 to 1.43)	0.96 (0.62 to 1.48)
	Overall	120/4955		
Treatment non-compliance	BMI >80th percentile	95/3531	1.40 (1.03 to 1.92)	1.38 (1.00 to 1.89)
	BMI ≤80th percentile†	96/5010	1.00 (reference)	1.00 (reference)
	Overall	191/8541		

*After accounting for age, sex, ethnicity, income, user type, global assessment of severity score, number of exacerbations in previous year, exposure to smoke, asthma-related comorbidities, triggers, % predicted FEV_1_, ICS rescue use and season.

†Reference category.

BMI, body mass index; FEV_1_, forced expiratory volume in 1 s; ICS, inhaled corticosteroids; LTRA, leukotriene receptor antagonist.

**Figure 2:**
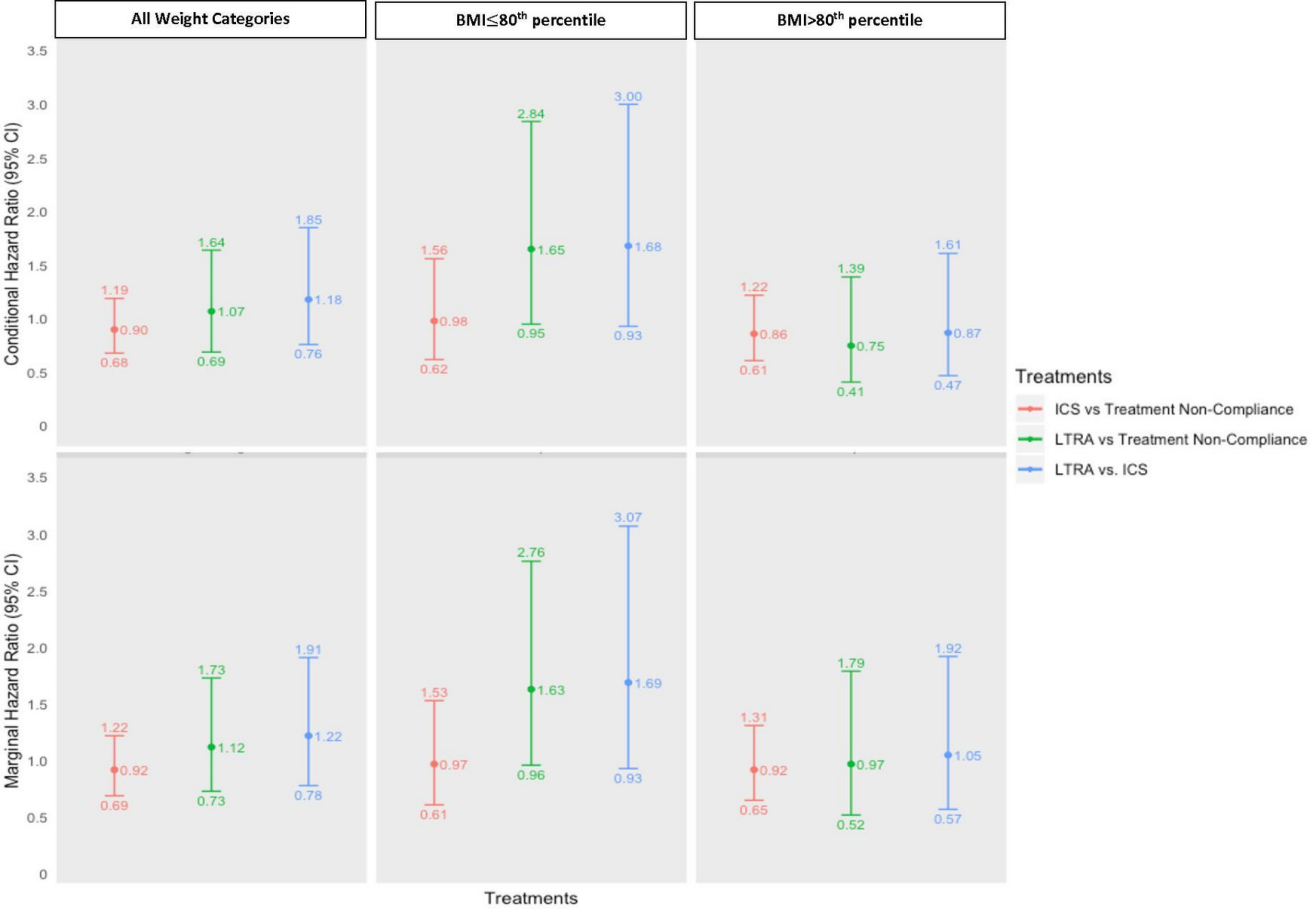
Conditional and marginal treatment HRs (95% CI) stratified by weight category and overall averages. The reference groups are treatment non-compliance (all weight categories), treatment non-compliance among those with a BMI ≤80th percentile (‘normal weight’) and treatment non-compliance among those with a BMI >80th percentile (‘overweight’). BMI, body mass index; ICS, inhaled corticosteroids; LTRA, leukotriene receptor antagonist.

**Figure 3:**
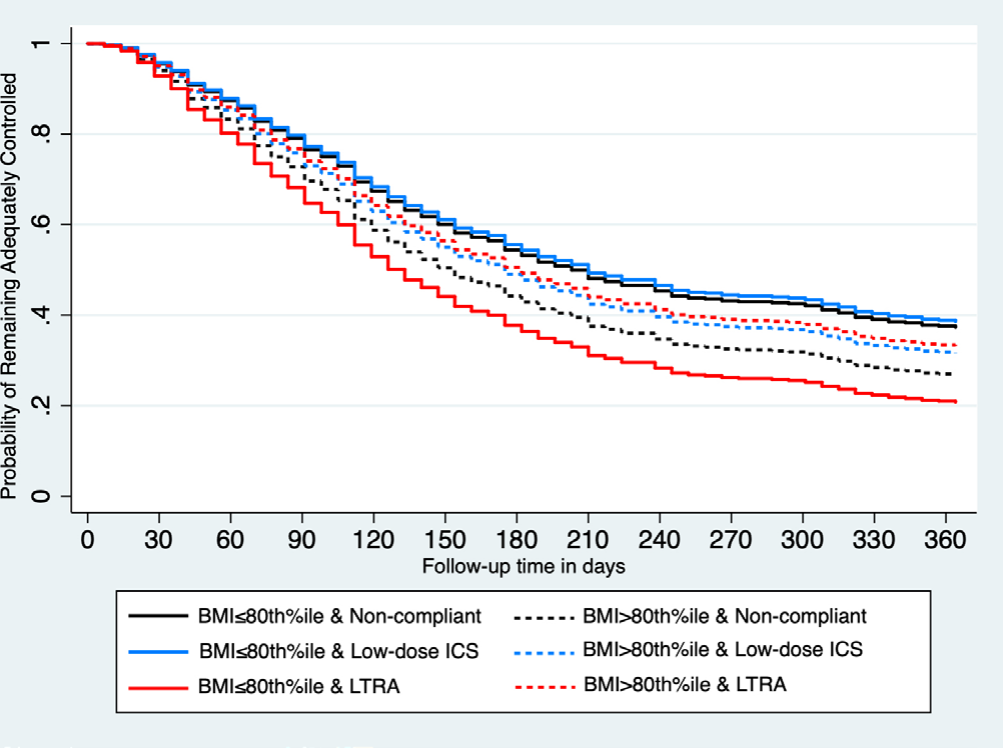
Marginal probability of not experiencing a management failure event, that is, remaining adequately controlled, for each weight status–treatment combination. BMI, body mass index; ICS, inhaled corticosteroids; LTRA, leukotriene receptor antagonist.

The HRs for different treatment comparisons stratified by normal weight and overweight subgroups are displayed graphically in figure 2. Survival curves for treatment and weight status category are illustrated in figure 3. Trends and conclusions remained consistent in incident and prevalent users, when restricting to patients with persistent asthma, and different methods of handling missing data (online supplemental tables e-2, e-3, and e-4).

## Discussion

In this cohort of children with asthma treated with Step-2 therapy, BMI percentile was associated with an overall increased risk of management failure. Our findings suggest a differential response to Step-2 maintenance therapies by weight status for LTRA. Whereas there was an overall greater risk of failure in children treated with LTRA, this was not observed in children with excess weight. No statistically significant interaction with weight was observed in children treated with low-dose ICS, although there was a trend for reduced response in children with higher BMIs.

Obesity is an established risk factor for poor control. We observed an overall 5%–6% increased risk of Step-2 monotherapy treatment failure for every 10-unit increase in BMI percentile in the main effects model and up to a 40% higher risk of failure in overweight children who were non-compliant to treatment in the analysis displaying HRs for different treatment–weight status categories. This is consistent with a recent meta-analysis of observational data including 46 070 children, which identified obesity as a significant determinant of asthma exacerbations (OR 1.17; 95% CI 1.03 to 1.34).^5^ Our results also suggest a differential response to Step-2 monotherapy by weight status. When compared with periods of treatment non-compliance and contrary to our hypothesis, no statistically significant treatment effect modification by weight was observed with ICS. However, whereas there was a trend towards overall poorer response for LTRA compared with low-dose ICS in the main effects model, a greater response to LTRA was observed at higher BMI percentiles when weight status was taken into account as a potential treatment response modifier in the interaction model. In other words, ICS and LTRA monotherapies seemed to be equally effective at prolonging time-to-management failure in children with a BMI >80th percentile, while low-dose ICS appeared to be, on average, more effective than LTRA in children with a BMI ≤80th percentile as graphically displayed.

To our knowledge, no previous studies have evaluated the differential response to LTRA relative to low-dose ICS by weight status in children, and the marginal structural models applied in this study are novel to paediatric asthma, although this approach has been previously implemented in a few adult asthma studies.^32–34^ Nevertheless, in a post-hoc analysis of a placebo-controlled randomised trial with 1041 children initiating low-dose ICS, Forno *et al* 10 reported that lung function and bronchodilator response were diminished by 0.4% and 0.25% for every 10% increase in BMI percentile, respectively. The authors did not evaluate response to LTRA for different BMIs. Yet, consistent with our findings, they demonstrated that normal-weight children initiating ICS experienced a significant reduction in the number of emergency department visits or hospitalisations for asthma during follow-up, whereas no improvement was observed in those with excess weight.^35^ Post-hoc analyses of adult randomized controlled trials also reported a differential effect of Step-2 therapy across weight status, although results were conflicting. Peters-Golden *et al* 36 noted that the effect of LTRA on asthma control days remained constant across weight categories, while that of low-dose ICS decreased with increasing BMI. Normal-weight adult patients randomised to low-dose ICS had a significantly greater percentage of asthma control days than those randomised to LTRA; however, this difference did not persist in patients with excess weight, consistent with our findings.^36^ Similarly, Sutherland *et al* 37 showed that patients with excess weight were more likely to experience a reduced response to ICS monotherapy in relation to exhaled nitric oxide levels when compared with their normal-weight counterparts, although without increasing the risk of exacerbation; in contrast, a reduced response to LTRA was not observed among those with higher BMI.^37^ In two other post-hoc analyses of RCTs, low-dose ICS was superior to LTRA with respect to improving FEV_1_, symptom scores and short-acting beta-2 agonist use across all weight categories, although it was suggested that response to both treatments may be attenuated at higher BMIs.^38 39^ Our paediatric study further supports ICS as a more effective option than LTRA in normal-weight children but not in overweight children, which aligns with Peters-Golden *et al*,^36^ where the response to LTRA was observed to increase with higher BMI.

There are several mechanisms underlying a possible differential response to LTRA and/or ICS by weight status in children. First, obesity has been shown to adversely affect pulmonary physiology by changing the elastic properties of the chest wall, leading to reduced forced vital capacity, FEV_1_ and peripheral airway diameter.^40^ In some children, the excess weight placed on the thoracic cage may inhibit lung expansion, which could lead to reduced absorption and deposition of inhaled therapies in the lower airways. Anderson and Lipworth^9^ showed that cortisol suppression was reduced in adults with asthma who were overweight when compared with their normal-weight counterparts, which they attributed to possible insufficient absorption and deposition of inhaled steroids.^9^ Perhaps, in children with higher weight-related lung compression, LTRA could offer an additional benefit over ICS as its efficacy is not affected by lung mechanics. Second, obese patients may exhibit an inflammatory process that is phenotypically different from the traditional Th2-dependent pathway—the latter underpins guidelines recommending low-dose ICS as a first-line Step-2 maintenance therapy. Moreover, some studies have suggested an airway inflammatory process partially mediated by leukotrienes, which may explain the increased response to LTRA at higher BMI percentiles in our study.^16 18^ Lastly, there is some evidence of shared genetic factors in the phenotypic variation of both obesity and asthma, including response to treatment.^41^ Polymorphisms in the B2-adrenergic and glucocorticoid receptor genes, as well as tumour necrosis factor-α, have been linked to asthma, obesity, and in some cases treatment response.^42–44^ Whatever the mechanism explaining the observed greater response to LTRA in those with excess weight, the findings suggest considering LTRA in patients with asthma with excess weight, if not as first line Step-2 therapy, at least as an alternative in those with suboptimal response to ICS, prior to step-up therapy.

The strengths of this study include specialist-diagnosed asthma (vs self-report), the availability of clearly recorded data on Asthma Centre physician prescriptions (type, dosage, and intended use of the asthma medication), as well as updated information on severity indicators throughout the follow-up period, which enabled the implementation of the marginal structural Cox model. In addition to a more intuitive interpretation, the marginal structural model generates a marginal HR that avoids biases arising from complicated cases of time-dependent confounding and non-collapsibility that are inherent to the conditional HR.^45 46^ Study findings should be interpreted in light of several limitations. Despite the inclusion of multiple covariates to adjust for differences between treatment groups, we suspect residual confounding by indication in this retrospective cohort analysis; indeed, children using their Step-2 therapies had similar or slightly higher HRs of management failure than those who were non-compliant to treatment, suggesting that the latter may have stopped using prescribed therapy because of milder symptoms compared with those who continued therapy. This residual confounding was minimised in the prevalent user subgroup results, since these children likely had a more persistent phenotype. Yet the consistency of findings in relation to the overall and treatment-specific effects by weight status on management failure in the subgroup analyses, irrespective of the marginal or conditional models, and the use of BMI as continuous or dichotomous variables, attests to the robustness of the results. We could not perform additional subgroup analyses based on age and sex due to our small sample sizes; however, we acknowledge the possibility that the effect of BMI percentile on time-to-management failure may vary across prepubertal, pubertal and postpubertal children due to developmental differences and hormones affecting adiposity and growth trajectory.^4^ Finally, because participants had to be enrolled in the Quebec public drug insurance programme, 49% of the original cohort were excluded, resulting in a reduced sample size and lower statistical power as well as an under-representation of children from higher income families who were privately insured. Moreover, participants were recruited from a tertiary care asthma clinic. Therefore, our findings may not be generalisable to children with asthma covered by private drug insurance, of higher socioeconomic status, or those not receiving specialised care.

In conclusion, our findings provide clinical insight into the association between weight status and treatment response in children initiated on Step-2 maintenance monotherapy. Children with higher BMI percentiles were overall more likely to experience early management failure despite Step-2 maintenance therapythan their normal-weight counterparts. Of interest, LTRA seems to achieve best response at higher BMI percentiles. While higher BMI status appears to be associated with a trend towards a reduced response to low-dose ICS, this association did not reach statistical significance. While addressing weight-reduction approaches to overcome challenges associated with obese-asthma, consideration may be given to LTRAs as an alternative Step-2 monotherapy in children with excess weight or at least in those with suboptimal response to low-dose ICS. However, given the limitations of this retrospective cohort study, further research is warranted to determine whether ICS and LTRA monotherapies are comparable in terms of prolonging time-to-management failure in higher risk obese children with asthma.

## References

[1] Kattan M, Kumar R, Bloomberg GR, et al Asthma control, adiposity, and adipokines among inner-city adolescents. J Allergy Clin Immunol 2010;125:584–92. 10.1016/j.jaci.2010.01.053 20226295 PMC3596816

[2] Quinto KB, Zuraw BL, Poon K-YT, et al The association of obesity and asthma severity and control in children. J Allergy Clin Immunol 2011;128:964–9. 10.1016/j.jaci.2011.06.031 21820711

[3] Jensen ME, Collins CE, Gibson PG, et al The obesity phenotype in children with asthma. Paediatr Respir Rev 2011;12:152–9. 10.1016/j.prrv.2011.01.009 21722842

[4] Jensen ME, Wood LG, Gibson PG Obesity and childhood asthma - mechanisms and manifestations. Curr Opin Allergy Clin Immunol 2012;12:186–92. 10.1097/ACI.0b013e3283508df5 22391755

[5] Ahmadizar F, Vijverberg SJH, Arets HGM, et al Childhood obesity in relation to poor asthma control and exacerbation: a meta-analysis. Eur Respir J 2016;48:1063–73. 10.1183/13993003.00766-2016 27587561

[6] Longo C, Bartlett G, Schuster T, et al The obese-asthma phenotype in children: an exacerbating situation? J Allergy Clin Immunol 2018;141:1239–49. 10.1016/j.jaci.2017.10.052 29382592

[7] Luder E, Melnik TA, DiMaio M Association of being overweight with greater asthma symptoms in inner city black and Hispanic children. J Pediatr 1998;132:699–703. 10.1016/S0022-3476(98)70363-4 9580773

[8] Black MH, Zhou H, Takayanagi M, et al Increased asthma risk and asthma-related health care complications associated with childhood obesity. Am J Epidemiol 2013;178:1120–8. 10.1093/aje/kwt093 23924576 PMC3857927

[9] Anderson WJ, Lipworth BJ Does body mass index influence responsiveness to inhaled corticosteroids in persistent asthma? Ann Allergy Asthma Immunol 2012;108:237–42. 10.1016/j.anai.2011.12.006 22469442

[10] Forno E, Lescher R, Strunk R, et al Decreased response to inhaled steroids in overweight and obese asthmatic children. J Allergy Clin Immunol 2011;127:741–9. 10.1016/j.jaci.2010.12.010 21377042 PMC3056233

[11] Global Initiative for Asthma Global strategy for asthma management and prevention, 2017.

[12] Shore SA Obesity and asthma: possible mechanisms. J Allergy Clin Immunol 2008;121:1087–93. quiz 94-5 10.1016/j.jaci.2008.03.004 18405959

[13] Lang JE, Obesity LJE Obesity, nutrition, and asthma in children. Pediatr Allergy Immunol Pulmonol 2012;25:64–75. 10.1089/ped.2011.0137 22768385 PMC3377949

[14] Rastogi D, Canfield SM, Andrade A, et al Obesity-associated asthma in children: a distinct entity. Chest 2012;141:895–905. 10.1378/chest.11-0930 21980061

[15] Leiria LOS, Martins MA, Saad MJA Obesity and asthma: beyond T(H)2 inflammation. Metabolism 2015;64:172–81. 10.1016/j.metabol.2014.10.002 25458831

[16] Jensen ME, Gibson PG, Collins CE, et al Airway and systemic inflammation in obese children with asthma. Eur Respir J 2013;42:1012–9. 10.1183/09031936.00124912 23349447

[17] Scott HA, Gibson PG, Garg ML, et al Airway inflammation is augmented by obesity and fatty acids in asthma. Eur Respir J 2011;38:594–602. 10.1183/09031936.00139810 21310876

[18] Telenga ED, Tideman SW, Kerstjens HAM, et al Obesity in asthma: more neutrophilic inflammation as a possible explanation for a reduced treatment response. Allergy 2012;67:1060–8. 10.1111/j.1398-9995.2012.02855.x 22686834

[19] British Thoracic Society and the Scottish Intercollegiate Guidelines Network British guideline on the management of asthma. A national clinical guideline, 2016.

[20] Lougheed MD, Lemiere C, Ducharme FM, et al Canadian Thoracic Society 2012 guideline update: diagnosis and management of asthma in preschoolers, children and adults. Can Respir J 2012;19:127–64. 10.1155/2012/635624 22536582 PMC3373283

[21] Lipworth BJ Leukotriene-receptor antagonists. Lancet 1999;353:57–62. 10.1016/S0140-6736(98)09019-9 10023966

[22] Anderson R, Theron AJ, Gravett CM, et al Montelukast inhibits neutrophil pro-inflammatory activity by a cyclic AMP-dependent mechanism. Br J Pharmacol 2009;156:105–15. 10.1111/j.1476-5381.2008.00012.x 19068077 PMC2697768

[23] Theron AJ, Steel HC, Tintinger GR, et al Cysteinyl leukotriene receptor-1 antagonists as modulators of innate immune cell function. J Immunol Res 2014;2014:1–16. 10.1155/2014/608930 PMC4058211 24971371

[24] Régie de l’assurance maladie du Québec Provisions of the public prescription drug insurance plan. Government of Quebec, 2015.

[25] Hernán MA, Alonso A, Logan R, et al Observational studies analyzed like randomized experiments: an application to postmenopausal hormone therapy and coronary heart disease. Epidemiology 2008;19:766–79. 10.1097/EDE.0b013e3181875e61 18854702 PMC3731075

[26] de Onis M, Onyango AW, Borghi E, et al Development of a WHO growth reference for school-aged children and adolescents. Bull World Health Organ 2007;85:660–7. 10.2471/BLT.07.043497 18026621 PMC2636412

[27] Robins JM, Hernán MA, Brumback B Marginal structural models and causal inference in epidemiology. Epidemiology 2000;11:550–60. 10.1097/00001648-200009000-00011 10955408

[28] Hernán MA The hazards of hazard ratios. Epidemiology 2010;21:13–15. 10.1097/EDE.0b013e3181c1ea43 20010207 PMC3653612

[29] Hernán MA, Brumback B, Robins JM Marginal structural models to estimate the causal effect of zidovudine on the survival of HIV-positive men. Epidemiology 2000;11:561–70. 10.1097/00001648-200009000-00012 10955409

[30] Hernan MA, Robins JM Causal inference: Part I. Boca Raton: Chapman & Hall/CRC, 2018.

[31] Richardson DB, Kaufman JS Estimation of the relative excess risk due to interaction and associated confidence bounds. Am J Epidemiol 2009;169:756–60. 10.1093/aje/kwn411 19211620 PMC3139969

[32] Kim C, Feldman HI, Joffe M, et al Influences of earlier adherence and symptoms on current symptoms: a marginal structural models analysis. J Allergy Clin Immunol 2005;115:810–4. 10.1016/j.jaci.2004.11.032 15806003

[33] Ali AK, Hartzema AG, Winterstein AG, et al Application of multicategory exposure marginal structural models to investigate the association between long-acting beta-agonists and prescribing of oral corticosteroids for asthma exacerbations in the clinical Practice Research Datalink. Value Health 2015;18:260–70. 10.1016/j.jval.2014.11.007 25773561

[34] Bédard A, Serra I, Dumas O, et al Time-dependent associations between body composition, physical activity, and current asthma in women: a marginal structural modeling analysis. Am J Epidemiol 2017;186:21–8. 10.1093/aje/kwx038 28453608 PMC6236938

[35] Forno E, Celedon JC The effect of obesity, weight gain, and weight loss on asthma Inception and control. Curr Opin Allergy Clin Immunol 2016. 10.1097/ACI.0000000000000339 PMC5545117 28030376

[36] Peters-Golden M, Swern A, Bird SS, et al Influence of body mass index on the response to asthma controller agents. Eur Respir J 2006;27:495–503. 10.1183/09031936.06.00077205 16507848

[37] Sutherland ER, Lehman EB, Teodorescu M, et al Body mass index and phenotype in subjects with mild-to-moderate persistent asthma. J Allergy Clin Immunol 2009;123:1328–34. 10.1016/j.jaci.2009.04.005 19501235 PMC2743451

[38] Sutherland ER, Camargo CA, Busse WW, et al Comparative effect of body mass index on response to asthma controller therapy. Allergy Asthma Proc 2010;31:20–5. 10.2500/aap.2010.31.3307 20167142

[39] Camargo CA, Sutherland ER, Bailey W, et al Effect of increased body mass index on asthma risk, impairment and response to asthma controller therapy in African Americans. Curr Med Res Opin 2010;26:1629–35. 10.1185/03007995.2010.483113 20429821

[40] Beuther DA, Weiss ST, Sutherland ER Obesity and asthma. Am J Respir Crit Care Med 2006;174:112–9. 10.1164/rccm.200602-231PP 16627866 PMC2662903

[41] Hallstrand TS, Fischer ME, Wurfel MM, et al Genetic pleiotropy between asthma and obesity in a community-based sample of twins. J Allergy Clin Immunol 2005;116:1235–41. 10.1016/j.jaci.2005.09.016 16337451 PMC2014783

[42] von Homeyer P, Schwinn DA Pharmacogenomics of β-adrenergic receptor physiology and response to β-blockade. Anesth Analg 2011;113:1305–18. 10.1213/ANE.0b013e31822b887e 21965354

[43] Hawkins GA, Amelung PJ, Smith RS, et al Identification of polymorphisms in the human glucocorticoid receptor gene (NR3C1) in a multi-racial asthma case and control screening panel. DNA Seq 2004;15:167–73. 10.1080/10425170410001704517 15497438

[44] Castro-Giner F, Kogevinas M, Imboden M, et al Joint effect of obesity and TNFA variability on asthma: two international cohort studies. Eur Respir J 2009;33:1003–9. 10.1183/09031936.00140608 19196817

[45] Austin PC The use of propensity score methods with survival or time-to-event outcomes: reporting measures of effect similar to those used in randomized experiments. Stat Med 2014;33:1242–58. 10.1002/sim.5984 24122911 PMC4285179

[46] Austin PC, Stuart EA Moving towards best practice when using inverse probability of treatment weighting (IPTW) using the propensity score to estimate causal treatment effects in observational studies. Stat Med 2015;34:3661–79. 10.1002/sim.6607 26238958 PMC4626409

