# Supplementary material for: Influence of weight status in the response to Step-2 maintenance therapies in children with asthma
Source: BMJ Open Respir Res. 2019 Apr 11;6(1):e000401. doi: 10.1136/bmjresp-2019-000401 (PMC6530505; doi:10.1136/bmjresp-2019-000401)
Supplement: Supplementary data [file bmjresp-2019-000401supp001.pdf]

SUPPLEMENT

a)

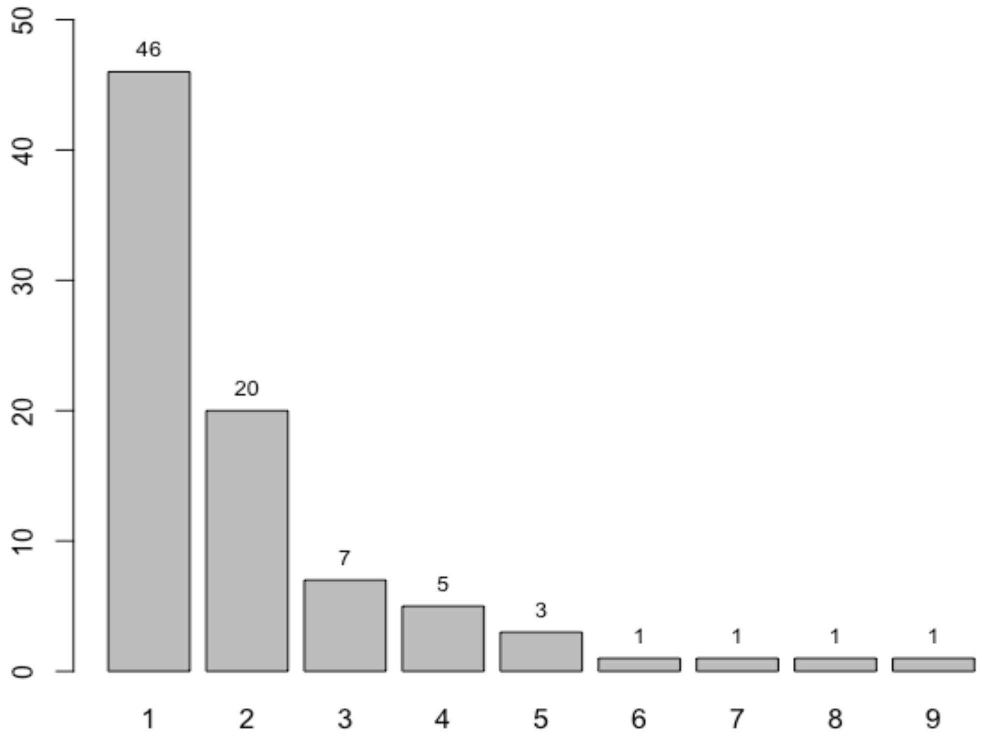

b)

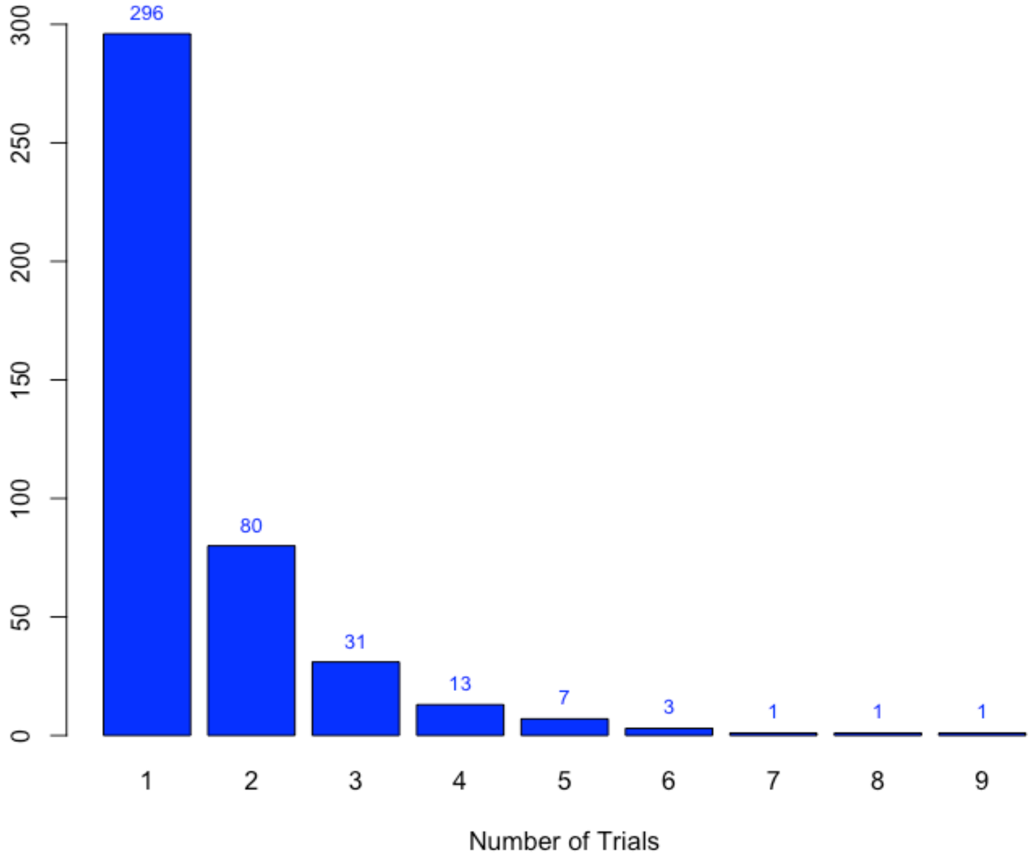

Figure e-1. Number of LTRA person-trials (a) and ICS person-trials (b) included in the cohort

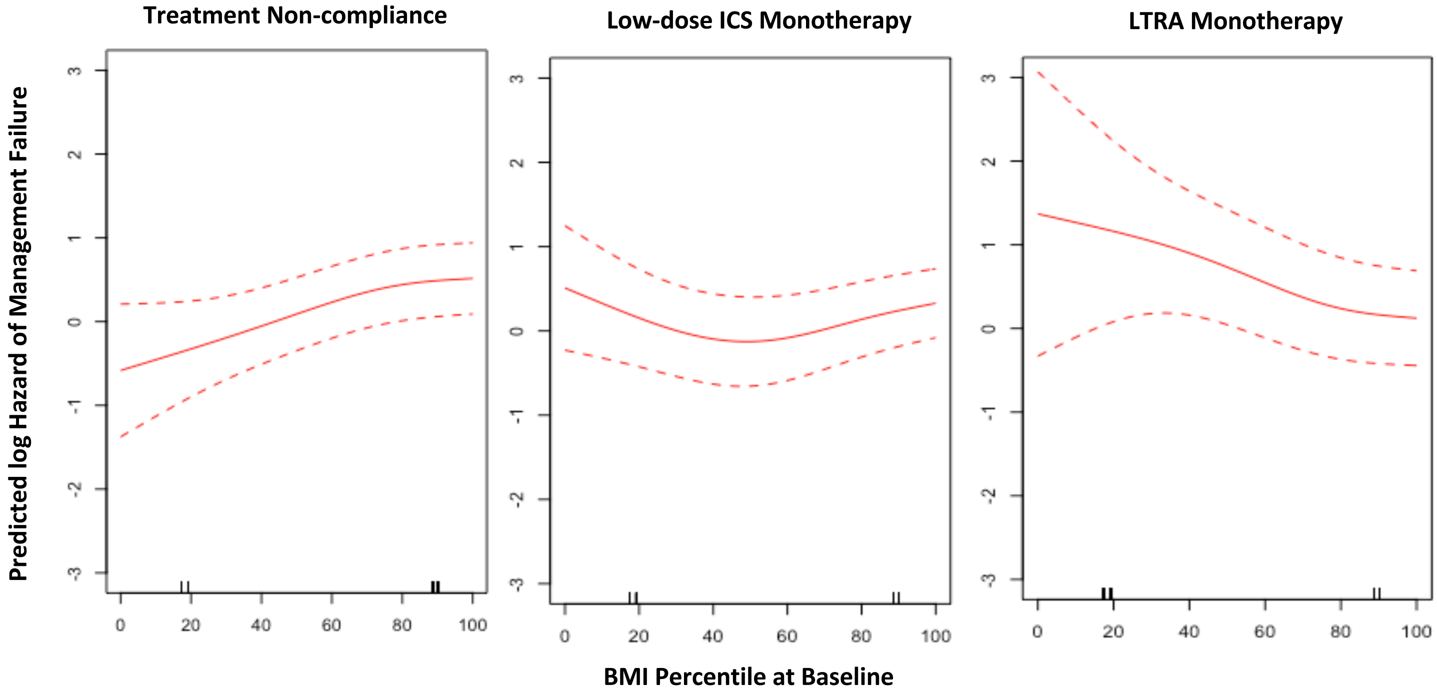

Figure e-2. The non-linear relationship between the predicted log hazards of management failure with BMI percentile at baseline for children not using their prescribed Step-2 therapy (treatment non-compliance) as well as those using low-dose ICS and LTRA monotherapies. The fit of the marginal model significantly improves with the 4-knot restricted cubic spline function for BMI percentile.
