# Supplementary material for: Influence of weight status in the response to Step-2 maintenance therapies in children with asthma
Source: BMJ Open Respir Res. 2019 Apr 11;6(1):e000401. doi: 10.1136/bmjresp-2019-000401 (PMC6530505; doi:10.1136/bmjresp-2019-000401)
Supplement: Supplementary data [file bmjresp-2019-000401supp002.pdf]

Table e-1a. Incidence Rates for Management Failure Events by Prescribed Treatment

Prescribed treatment at index visit	Visit no.	No. of patients	No. of failure events/trial-weeks	Incidence rate (per 100 trial-weeks)
LTRA Monotherapy	1	46	28/1369	2.05
	2	20	13/594	2.20
	3+	19	12/548	2.20
	All	85	53/2511	2.11
Low-dose ICS Monotherapy	1	296	189/8076	2.34
	2	80	52/2283	2.28
	3+	56	45/1569	2.87
	All	433	286/11928	2.40

Table e-1b. Incidence Rates for Management Failure Events by Treatment Use

Treatment used during follow-up	Visit no.	No. of patients*	No. of failure events/trial-weeks	Incidence rate (per 100 trial-weeks)
LTRA Monotherapy	1	38	9/459	1.96
	2	15	10/215	4.65
	3+	19	9/359	2.51
	All	72	28/1033	2.71
Low-dose ICS Monotherapy	1	218	80/3366	2.38
	2	60	21/844	2.49
	3+	40	19/745	2.55
	All	318	120/4955	2.42
Treatment Non-compliance	1	278	128/5686	2.25
	2	86	34/1833	1.85
	3+	63	29/1022	2.84
	All	427	191/8541	2.24

*Since the treatment classification during follow-up is dynamic, e.g. patients may be on and off of their prescribed treatments, the number of patients for each treatment-trial category were not mutually exclusive.

Table e-2. Missing data sensitivity analysis: Comparison of effects with multiple imputations (original analysis), restricting to children with non-missing BMI, or complete cases

Models	Marginal Average Hazard Ratio (95% CI)[a]		
	Original Analysis (N=518)	Non-missing BMI (N=512)	Complete Cases (N=330)
**Main Effects Model**			
BMI percentile (for every 10-units)	1.05 (1.01,1.10)	1.05 (1.01,1.10)	1.08 (1.02,1.14)
ICS vs. treatment non-compliance	0.93 (0.70,1.23)	0.93 (0.70,1.23)	0.87 (0.63,1.20)
LTRA vs. treatment non-compliance	1.11 (0.72,1.70)	1.11 (0.72, 1.70)	1.00 (0.60,1.67)
**Model with Interaction Term**			
BMI percentile (for every 10-units)	1.08 (1.02,1.15)	1.08 (1.02,1.15)	1.11 (1.04,1.19)
ICS vs. treatment non-compliance	1.37 (0.61,3.10)	1.35 (0.61,3.03)	1.26 (0.53,3.00)
LTRA vs. treatment non-compliance	4.62 (1.27,16.88)	4.60 (1.25,16.95)	6.36 (1.71,23.71)
ICS BMI percentile*	0.95 (0.86,1.04)	0.95 (0.86,1.05)	0.95 (0.85,1.06)
*LTRA*BMI percentile*	0.83 (0.70,0.99)	0.84 (0.70,0.99)	0.80 (0.66,0.96)
**LRT p-value[b]**	<.001	<.001	0.031
**RERI ICS*BMI percentile[c]**	-0.06 (-0.16,0.04)	-0.05 (-0.14,0.05)	-0.04 (-0.12,0.04)
**RERI LTRA*BMI percentile[c]**	-0.52 (-1.76,0.71)	-0.52 (-1.74,0.71)	-1.34 (-3.80,1.13)

[a] After accounting for age, sex, ethnicity, income, user type, global assessment of severity score, number of exacerbations in previous year, exposure to smoke, asthma-related comorbidities, triggers, % predicted FEV1, ICS rescue use, and season. Body Mass Index (BMI); Inhaled Corticosteroids (ICS); Leukotriene Receptor Antagonists (LTRA). The original analysis refers to multiply imputed datasets, the non-missing BMI analysis refers to restricting the sample to only those children with non-missing BMI, and the complete cases analysis refers to restricting the sample to only those children with no missing data.

[b] The Likelihood ratio test (LRT) assesses improved goodness of fit when comparing nested models (the model with interaction terms vs. the main effects model); a p-value <0.05 indicates a statistically significant improved fit, i.e. explaining a greater proportion of the variance in the outcome, and the likely presence of effect measure modification on the multiplicative scale.

[c] The relative excess risk due to interaction (RERI) = $HR_{Therapy*BMI} - HR_{Therapy} - HR_{BMI} + 1$; a negative RERI can be interpreted as the hazard reduction due to interaction on the additive scale (sub-additivity), adjusted for measured confounders.

Table e-3. Sensitivity analysis: conditional and marginal model estimates of time-to-management failure stratified by user type

Models	Average Hazard Ratio (95% CI)*	
	**Conditional**	**Marginal**
**Main Effects Model**		
Incident Users		
BMI percentile (for every 10-units)	1.03 (0.96, 1.12)	1.03 (0.96, 1.11)
ICS vs treatment non-compliance	0.98 (0.63, 1.52)	0.98 (0.63, 1.52)
LTRA vs. treatment non-compliance	2.82 (1.26, 6.29)	2.53 (1.04, 6.13)
LTRA vs. ICS	2.89 (1.18, 7.06)	2.50 (0.95, 6.59)
Prevalent Users		
BMI percentile (for every 10-units)	1.05 (1.00, 1.11)	1.05 (0.99, 1.11)
ICS vs treatment non-compliance	0.82 (0.57, 1.17)	0.86 (0.60, 1.24)
LTRA vs. treatment non-compliance	0.86 (0.50, 1.46)	1.02 (0.64, 1.63)
LTRA vs. ICS	1.05 (0.62, 1.79)	1.18 (0.72, 1.95)
**Model with Product Term**		
Incident Users		
BMI percentile (for every 10-units)	1.07 (0.98, 1.17)	1.07 (0.98, 1.18)
ICS vs treatment non-compliance	1.90 (0.61, 5.94)	2.01 (0.63, 6.46)
LTRA vs. treatment non-compliance	7.46 (1.24, 44.85)	6.00 (1.13, 31.76)
ICS BMI percentile*	0.91 (0.78, 1.05)	0.90 (0.77, 1.05)
*LTRA*BMI percentile*	0.87 (0.67, 1.13)	0.88 (0.70, 1.11)
**LRT p-value†**	0.0052	0.0051
**RERI ICS*BMI percentile‡**	-0.12 (-0.43, 0.18)	-0.14 (-0.47, 0.20)
**RERI LTRA*BMI percentile‡**	-0.57 (-3.01, 1.89)	-0.39 (-2.04, 1.25)
Prevalent Users		
BMI percentile (for every 10-units)	1.09 (1.01, 1.18)	1.07 (0.99, 1.16)
ICS vs treatment non-compliance	1.24 (0.43, 3.58)	1.03 (0.36, 2.98)
LTRA vs. treatment non-compliance	7.66 (0.99, 59.15)	5.39 (0.62, 46.79)
ICS BMI percentile*	0.94 (0.83, 1.07)	0.98 (0.85, 1.11)
*LTRA*BMI percentile*	0.77 (0.59, 0.99)	0.82 (0.62, 1.07)
**LRT p-value†**	<0.0001	0.031
**RERI ICS*BMI percentile‡**	-0.06 (-0.18, 0.07)	-0.03 (-0.12, 0.07)
**RERI LTRA*BMI percentile‡**	-1.35 (-5.46, 2.75)	-0.75 (-3.43, 1.93)

*After accounting for age, sex, ethnicity, income, global assessment of severity score, number of exacerbations in previous year, exposure to smoke, asthma-related comorbidities, triggers, %predicted FEV1, ICS rescue use, and season. Body Mass Index (BMI); Inhaled Corticosteroids (ICS); Leukotriene Receptor Antagonists (LTRA).

†The Likelihood ratio test (LRT) assesses improved goodness of fit when comparing nested models (the model with product term vs. the main effects model); a p-value <0.05 indicates a significantly improved fit, i.e. explaining a greater proportion of the variance in the outcome, and the likely presence of effect measure modification on the multiplicative scale.

‡The relative excess risk due to interaction (RERI) = $HR_{Therapy*BMI} - HR_{Therapy} - HR_{BMI} + 1$; a negative RERI can be interpreted as the hazard reduction due to interaction on the additive scale (sub-additivity), adjusted for measured confounders.

Table e-4. Sensitivity analysis: conditional and marginal model estimates of time-to-management failure for only those with persistent asthma

Models	Average Hazard Ratio (95% CI)*	
	Conditional	Marginal
**Main Effects Model**		
Incident Users		
BMI percentile (for every 10-units)	1.08 (1.01, 1.14)	1.07 (1.01, 1.14)
ICS vs treatment non-compliance	0.96 (0.67, 1.37)	0.95 (0.66, 1.36)
LTRA vs. treatment non-compliance	1.07 (0.59, 1.94)	1.17 (0.65, 2.09)
**Model with Product Term**		
BMI percentile (for every 10-units)	1.11 (1.02, 1.20)	1.09 (1.00, 1.18)
ICS vs treatment non-compliance	1.24 (0.44, 3.51)	1.00 (0.34, 2.97)
LTRA vs. treatment non-compliance	10.82 (3.06, 38.25)	7.22 (1.48, 35.16)
ICS BMI percentile*	0.97 (0.85, 1.09)	0.99 (0.87, 1.13)
*LTRA*BMI percentile*	0.76 (0.64, 0.90)	0.80 (0.65, 0.99)
**LRT p-value†**	<0.0001	0.0001
**RERI ICS*BMI percentile‡**	-0.02 (-0.10, 0.06)	0.00 (-0.07, 0.06)
**RERI LTRA*BMI percentile‡**	-1.85 (-5.29, 1.60)	-1.01 (-3.62, 1.61)

*After accounting for age, sex, ethnicity, income, user type, global assessment of severity score, number of exacerbations in previous year, exposure to smoke, asthma-related comorbidities, triggers, %predicted FEV1, ICS rescue use, and season. Body Mass Index (BMI); Inhaled Corticosteroids (ICS); Leukotriene Receptor Antagonists (LTRA).

†The Likelihood ratio test (LRT) assesses improved goodness of fit when comparing nested models (the model with product term vs. the main effects model); a p-value <0.05 indicates a significantly improved fit, i.e. explaining a greater proportion of the variance in the outcome, and the likely presence of effect measure modification on the multiplicative scale.

‡The relative excess risk due to interaction (RERI) = $HR_{Therapy*BMI} - HR_{Therapy} - HR_{BMI} + 1$; a negative RERI can be interpreted as the hazard reduction due to interaction on the additive scale (sub-additivity), adjusted for measured confounders.
